# Association of *MicroRNA 196a* and *499 Polymorphisms* with Development of Cirrhosis and Hepatocellular Carcinoma Post-HCV Infection in Egyptian Patients

**DOI:** 10.31557/APJCP.2019.20.11.3479

**Published:** 2019

**Authors:** Asmaa Mohamed Fteah, Asmaa Ismail Ahmed, Nehad Ahmed Mosaad, Mona Mohamed Hassan1, Sherif Hamdy Mahmoud

**Affiliations:** 1 *Department of Clinical and Chemical Pathology, Theodor Bilharz Research Institute, *; 2 *Department of Clinical and Chemical Pathology, Kasr Al-Ainy, *; 3 *Department of Endemic Medicine, Liver Unit, Faculty of Medicine, Cairo University, Egypt. *

**Keywords:** Hepatocellular carcinoma, Hepatitis C Virus, MiR-196a2 C>T, MiR-499 A>G, Real Time PCR

## Abstract

Hepatocellular carcinoma (HCC) is the commonest primary tumor of the liver. Chronic HCV infection is the leading cause of end-stage liver disease, HCC and liver-related death in Egypt. Single nucleotide polymorphisms (SNPs) in microRNAs were reported to increase susceptibility to tumorigenesis; affect prognosis and as promising biomarkers in virus-host interactions. This study was conducted to investigate the role of genetic variants of* miR-196a2 (rs 11614913) *C>T and *miR-499 (rs 3746444) *A>G in the development of cirrhosis and HCC in Egyptian HCV infected patients. Genotyping of the candidate SNPs was performed by Real Time PCR in 75 HCV-related HCC patients, 75 cirrhotic patients on top of HCV and 75 healthy controls. There was significant difference in *miR-499 (rs3746444)* genotypes frequency between the three studied groups as the GG genotype was significantly lower in HCC cases than other groups (P = 0.009) while the combined miR-499 (AA+AG) genotypes were significantly higher in HCC cases than other groups (P = 0.005). Also a significant difference was found in* miR-499* genotypes frequency when compared between HCC and cirrhosis groups as the GG genotype was significantly lower in HCC cases than cirrhosis group (P = 0.006) while the combined* miR-499 (AA+AG)* genotypes were significantly higher in HCC cases than in cirrhosis group (P = 0.003) [OR (95% CI) = 0.131 (0.028-0.601)]. The frequency of the G allele was significantly lower in HCC than other groups (P = 0.024) and significantly lower in HCC than normal group (P = 0.006) [OR (95%CI) = 0.501 (0.304-0.825)]. For *miR-196a2 (rs11614913) C>T* polymorphisms, no significant association was found with HCC risk. Our study concluded that the G allele of miR-499 is associated with lower risk of HCV related HCC development. No significant association of *miR-196a2 (rs 11614913)*, genotypes or alleles with risk for HCC development, could be detected.

## Introduction

Hepatocellular carcinoma is the fifth most common tumor worldwide and the second most common cause of cancer-related death (Choo et al., 2016). According to Globocan (2012), HCC is the first most common cancer in men and the second most common cancer in women in Egypt. Chronic HCV infection is considered an independent risk factor for liver cirrhosis and HCC (Mohd Hanafiah et al., 2013). The incidence of HCC is significantly more prevalent in rural residents and in patients with previous history of Schistosomiasis and/or blood transfusion. According to Egyptian Demographic Health Survey (EDHS) 2015; the seroprevalence of HCV was 10% in the age groups 15–59 years in Egypt (Gomaa et al., 2017). HCC management requires a multidisciplinary approach because of the wide heterogenecity in clinical manifestations, differences in biologic behaviour, different causes of chronic liver disease and different therapeutic protocols (Abdelaziz et al., 2014). 

MicroRNAs are small single stranded non-coding RNAs; transcribed from DNA sequences into primary miRNAs transcripts and processed into precursor and mature miRNAs. In most cases, miRNAs interact with the 3′ UTR of target mRNAs to suppress their expression (Ha and Kim, 2014). MicroRNAs shuttled between different subcellular compartments to regulate the rate and efficiency of translation and transcription (Makarova et al., 2016). MicroRNAs are critical for normal development and involved in a variety of biological processes (Fu et al., 2013). In addition, extracellular miRNAs serve as signaling molecules to mediate cell-cell communications (Wang et al., 2016; Huang, 2017). Aberrant expression of miRNAs is associated with a variety of human diseases (Paul et al., 2018). Many miRNAs are deregulated in HCC as they may act as oncogenes or as tumor suppressors (Lujambio and Lowe, 2012). While other several miRNAs act as key players in hepatitis C virus-host interactions (Cheng et al., 2012). HCV modulates the expression of miRNA leading to hepatocyte growth towards tumorigenesis through regulation of various signaling pathways (Giordano and Columbano, 2013).

It is expected that sequence variations such as SNPs at miRNA binding sites may affect the expression of miRNA targets. Such SNPs associated with deregulation of the expression of oncogenes or tumour suppressor genes might contribute to tumourgenesis (Srivastava et al., 2017). Increasing evidence has indicated that miRNAs play important roles in carcinogenesis through post-transcriptional gene silencing (Wang et al, 2018).

Therefore, the aim of our work was to evaluate *MicroRNA 196a2 (rs 11614913)* and *499 (rs 3746444) SNPs *as potential biomarkers for liver cirrhosis and HCC development in Egyptian patients following HCV infection, and their correlation with clinicopathological features.

## Materials and Methods

*Ethical approval*

Before commencing the study; approval was obtained from the Institutional Review Board of Theodor Bilharz Research Institute (FWA 00010609), and written informed consent was obtained from each participant, in accordance with the ethical standards of the ethics committee of our hospital and with the 1975 Helsinki declaration and its later amendments.

*Study population*

This case-control study was carried out from April 2017 to August 2018 on a total number of 225 age and sex-matched subjects, who were divided into three groups. Group I: included 75 patients with HCC on top of HCV infection and liver cirrhosis. Diagnosis was done according to European Association for the study of the liver (EASL) guidelines (Llovet et al., 2012). Group II: included 75 patients with liver cirrhosis on top of HCV infection as a pathological control group. They were diagnosed by abdominal ultrasound and evidence of chronic infection with HCV. All HCV-related HCC and cirrohsis patients were recruited from the Tropical Medicine departments at Kasr al Ainy hospital, Cairo University and Theodor Bilharz Research Institute. Group III: included 75 apparently healthy subjects, randomly selected from individuals attending the outpatient clinic for other reasons. Healthy control group participants were selected with normal liver function tests, negative HBsAg and HCV Ab tests and no history of bilharziasis, alcohol abuse and diabetes.

The exclusion criteria were patients who had recurrent or secondary tumors, history of past malignancies, other liver diseases, HBsAg positive patients, or had inflammatory conditions as spontaneous bacterial peritonitis, inflammatory bowel diseases or systemic sepsis and those who were below 18 years old.

Hepatocellular carcinoma and cirrhotic patients were subjected to full history taking, complete clinical examination, assessment of liver functions using Child-Pugh score and stratification of patients according to the severity of the disease in an objective and continuous ranking scale by the Model of End-stage Liver Disease (MELD) score.

*Laboratory investigations*

The laboratory status of HCC and cirrhotic patients, was assessed by routine liver and kidney function tests including serum total bilirubin, direct bilirubin, alanine aminotranseferase (ALT), aspartate aminotransaminase (AST), total protein, serum albumin, alkaline phosphatase (ALP), serum urea and creatinine. They were assayed spectrophotometrically by the automated analyzer Beckman Coulter AU 480 (Beckman Coulter, Inc.: CA, USA; supported by BM-Egypt, Cairo, Egypt).

- Serum AFP level was assayed using chemiluminescence method by the automated analyzer ADVIA Centaur-CP (Siemens Healthcare Diagnostics Inc.: NY, USA; supported by Siemens Healthineers, Cairo, Egypt). 

- Serum HBsAg and HCV Ab assayed using enzyme immunoassay (Human GmbH: Germany; supported by GammaTrade Company, Cairo, Egypt).

- Complete blood picture (CBC), was done on automated cell counter Beckman Coulter AcT Diff. (Beckman Coulter, Inc.: CA, USA; supported by BM-Egypt, Cairo, Egypt).

- Prothrombin concentration (PC) and International Normalized Ratio (INR), were assayed by the automated coagulometer Dade-Behring (Siemens Healthcare Diagnostics Inc.: NY, USA; supported by Siemens Healthineers, Cairo, Egypt). 

While the normal control group was investigated for routine liver and kidney function tests and hepatitis markers.

*Analysis of microRNA-SNPs*

*DNA Extraction*

Two milliliters of whole blood samples were collected into a sterile EDTA vacutainer tube and stored at - 20ºC. Extraction of genomic DNA from peripheral blood leucocytes of EDTA anticoagulated blood was done using the Thermo Scientific GeneJET Whole Blood Genomic DNA Purification Mini Kit (Thermo Fisher Scientific Inc.: CA, USA; provided by Clinilab, Cairo, Egypt). 

*Genotyping*

Real-Time PCR with sequence-specific primers was used to define the *miR-499 (rs 3746444) (A>G) *and *miR-196a2 (rs 11614913) (C>T)* single nucleotide polymorphisms. Amplification of the extracted DNA and Real-time PCR allelic discrimination assay was designed using Taq-Man SNP Genotyping Assays (Applied Biosystems§) according to the protocol proposed by Kristiansen et al., 2001. The thermal cycling conditions was programmed as follows: 95°C for 10 minutes, 50 cycles of 92°C for 10 seconds and 60°C for 1 minute. Data analysis for allele discrimination was performed with the Applied Biosystems step one Real-Time PCR System software (Applied Biosystems, Foster City: CA, USA; provided by Clinilab, Cairo, Egypt). Appropriate controls were included in each genotyping run for both micro-RNAs to monitor contamination of reagents.

* All laboratory tests were performed in the Chemical Pathology Unit, Theodor Bilharz Research Institute; while molecular studies were performed in the Chemical Pathology Department, Cairo University Hospitals.

*Statistical analysis*

Data were coded and entered using the software SPSS for Mac, release 24 (IBM Corporation, Armonk, NY, USA). Parametric data were summarized as mean±SD, non parametric data as median and percentiles for quantitative variables, while frequency and percentages were used for qualitative variables. Comparison between groups was done using Chi square test and Fischer exact test for qualitative variable, t test and non-parametric Mann-Whitney U test were used to compare two groups. For comparing three groups; Anova and Kruskal Wallis tests were used. The risk estimate was done using odd’s ratio (OR) and their 95% confidence intervals (CI) were calculated to estimate the strength of the association between single nucleotide polymorphisms and the study population (HCC patients, cirrhosis and normal control groups). A p value < 0.05 was considered significant (Knapp and Miller, 1992).

## Results

*The clinical and laboratory data*

The demographic, clinical, laboratory and radiologic data of the three studied groups are demonestrated in Table 1.

A significant association could be detected between positive family history of HCC and the development of HCC in comparison to liver cirrhosis. There are 2 cirrhotic patients who have positive family history but did not develop HCC and 32% of HCC patients had positive history compared to only 2.7% of cirrhotic patients. Bilharziasis was found to be significantly higher in HCC group in comparison to liver cirrhosis.

Out of the laboratory tests conducted on all patients; hemoglobin, creatinine, albumin, AST, total bilirubin and direct bilirubin showed significant difference between the three studied groups. Total protein showed significant difference between HCC and cirrhosis groups on one hand and cirrhosis and normal control groups on the other hand; but no significant difference between HCC and normal control groups. Platelet count, prothrombin concentration, INR and ALT showed significant difference between HCC and normal control groups on one hand and cirrhosis and normal groups on the other hand; but no significant difference between HCC and cirrhosis groups. Serum AFP was significantly higher among HCC patients compared to the cirrhotic patients.

*Frequency distribution of microRNA 499*

The frequency distribution of *miR-499 (rs3746444) *genotypes, alleles and Odd’s Ratio among the studied groups and its association with HCV-related HCC are shown in Table 2, while Table 3 compares between (AA+AG) and GG genotypes in HCC and cirrhosis group as regards the laboratory data. 

There was a significant difference in the frequency of *miR-499 (rs3746444) *genotypes between the three studied groups as the GG genotype was significantly lower in HCC cases than other groups (P = 0.009) while the combined *miR-499(AA+AG)* genotypes were significantly higher in HCC cases than other groups (P = 0.005). When the genotypic distribution of *miR-499 (rs3746444)* compared between HCC and cirrhosis groups; the GG genotype was found to be significantly lower in HCC cases than cirrhosis group (P = 0.006), while the combined miR-499 (AA+AG) genotypes were significantly higher among HCC cases than in cirrhosis group (P = 0.003) [OR = 0.131, 95% CI = 0.028 - 0.601]. There was a significant difference in* miR-499 (rs3746444) *genotypes frequency when compared between HCC patients and normal control group as the combined *miR-499 (AA+AG)* genotypes were significantly higher in HCC cases than in normal control group [OR = 0.119, 95% CI = 0.026 - 0.847] and (P = 0.002). 

Study of allele frequency of* miR-499 (rs 3746444) *among the three studied groups revealed that the G allele was significantly lower in HCC cases than other groups (P = 0.024) and significantly lower in HCC cases than normal control group [OR (95%CI) = 0.501 (0.304-0.825)] and (P = 0.006). 

The median of AST, ALT, ALP, total bilirubin and direct bilirubin levels was significantly lower in patients with GG genotype than those with combined (AA+AG) genotypes.

*Frequency distribution of microRNA 196a2*

Table 4 illustrates the frequency distribution of *miR-196a2 (rs11614913)* genotypes and alleles among the three studied groups.

Regarding *miR-196a2 (rs 11614913)*, no significant association could be detected among the three studied groups (P = 0.344); and collapse of TT and CT genotypes didn’t yield significant association (P = 0.144). Also, the study of allele frequency of* miR-196a2* among the three studied groups did not show a significant association as well (P = 0.152).

## Discussion

The *miR-499 (rs3746444) A>G* polymorphism was reported to promote carcinogenesis, which is supported by the results of our study that demonstrated a statistically significant difference in the frequencies of *miR-499 (rs3746444)* genotypes when compared between the three studied groups as the GG genotype was significantly lower in HCC cases than other groups, while the combined *miR-499 (AA+AG)* genotypes were significantly higher in HCC cases than other groups. A significant increase in the combined *miR-499 (AA+AG)* genotypes, among HCC cases than in cirrhosis group; indicating that cirrhotic patients harboring GG genotype are 7.6 times (1.7-35.2) more protected than those harboring (AA/AG) genotypes to progress to HCC. While the combined miR-499 (AA+AG) genotypes were significantly higher in HCC cases than in normal control group; finding that normal individuals carrying GG genotype are 8.4 times (1.8-38.3) more protected than those with (AA/AG) genotypes to develop HCC. As well as the G allele of *miR-499 (rs 3746444)* found to be significantly lower in HCC cases than other groups. 

The median of AST, ALT, ALP, total bilirubin and direct bilirubin levels was significantly lower in patients with GG genotype than those with combined (AA+AG) genotypes. These results suggest that miR-499 (rs3746444) polymorphisms may have functional role in the development of HCC. 

A case-control study reported the association between miR-499 (A/G) genotypes and HCC; the authors demonstrated a significant association of *miR-499 (rs3746444) GG* genotype with a decreased risk of HCC as compared with the* miR-499 AA* genotype [adjusted OR (AOR) = 0.74, 95% CI = 0.24 - 0.96], and a 0.45-fold decreased HCC risk in a recessive model, as well as a significant decrease in the frequency of the G allele among HCC cases compared to the control group (Zou and Zhao, 2013). Furthermore, a significant decrease in the frequency of the AG genotype was reported among HCC patients when compared to the control subjects, [AOR = 0.74, 95% CI = 0.24 - 0.96] and (P < 0.05) (Shan et al., 2013). These results go hand by hand with our study; suggesting that the carriage of G allele might decrease the risk of HCC. 

On contrary, it was indicated that subjects carrying the *rs 3746444 AG* or *GG* genotypes showed a significantly increased susceptibility to HCC [AOR = 2.84, 95% CI = 1.88 - 4.30] (Chu et al., 2014). 

Up to our knowledge, the only study conducted on Caucasian population found no significant difference in genotypic or allelic distributions between HCC patients and controls. However, their patients were 60% HBsAg positive and only 24% anti-HCVAb positive. Another discrepancy in their study compared to ours is the high presentation for GG genotype (40% of cases and 37% of controls) and G allele in their participants (Akkiz et al., 2011). Other studies on Chinese population failed to find a significant association between miR-499 polymorphisms and the development of HCC (Hao et al., 2013;Yan et al., 2015; Li et al., 2015). Additionally, a subgroup analysis by ethnicity showed no correlation between *miR-499 (A/G) *genotypes and HCC in Caucasian, Asian and Chinese using all genetic models in a huge meta-analysis study (Zheng et al., 2017).

In our study, we included 150 chronic liver disease patients divided into HCC and cirrhotic patients, the inclusion of different stages of liver illness with gradually increasing matched results, can be considered a point of strength. In addition, the method of assay is more accurate than RFLP, the ethnicity difference from Chinese population, and more important, the results of liver function tests supported the results of genotyping methods.

Regarding* miR-196a2 (rs 11614913)*, no significant association could be detected among the three studied groups (P = 0.344); and collapse of TT and CT genotypes didn’t yield significant association (P = 0.144). Also, the study of allele frequency of *miR-196a2* among the three studied groups did not show a significant association as well (P = 0.152).

In concordance to our results; a significant difference in the frequency of *miR-196a2 (rs11614913) *genotypes couldn’t be found among HCC cases when compared to the control group (Chu et al., 2014). While it was reported that the genotype distributions between HCC cases and normal control subjects showed significant difference for *miR-196a2 (C>T)*; finding that *miR-196a2 CC* genotype and C allele have an important role in HCC risk in Chinese, especially in patients with HBV infection (Hao et al., 2013). 

Also a case-control study indicated that TT genotype and T allele of* miR-196a2 (rs11614913)* carried a 2.29-fold (95% CI = 1.30- 4.05) and 1.6-fold (95% CI = 1.11-2.32) increased risk of HCC when compared with CC genotype, respectively (Li et al., 2015). While other findings showed that individuals carrying the TC and CC genotypes of *miR-196a2 (rs11614913)* to be associated with an elevated risk of HCC compared to the TT genotype (p < 0.001) and AOR (95% CI) were [1.50 (1.03-2.17)] and [2.86 (1.60-5.16)], respectively. Moreover, the TC+CC genotype was correlated with an increased risk of HCC (P = 0.003) and (OR=1.69, 95% CI=1.19-2.41) compared to the TT genotype (Yan et al., 2015).

The discrepancy between the results of our study and those of others may be attributed to the large ethnic and geographic variabilities in the incidence of HCC among different populations and the different HCV genotypes other than genotype 4 which represents about 90% of cases in Egypt (Frank et al., 2000).

Further large-scale studies are suggested considering gene/gene and gene/environment interactions and ethnicity of patients, which may explain the discrepancy between the results of different studies.

In conclusions, there was a significant difference in *miR-499 A>G (rs3746444)* genotypes frequency when compared between the three studied groups. The G allele of *miR-499 A>G (rs3746444)* was significantly lower in HCC cases than other groups. The current study suggested that *miR-499 A>G (rs3746444)* gene polymorphisms may exhibit an influence on HCC susceptibility in Egyptian patients. For *miR-196a2 C>T (rs11614913)* polymorphisms, although the CC homotype was found to be more prevalent than TT homotype in our studied groups but this didn’t yield a significant association between miR-196 polymorphisms with risk for HCC development.

**Table 1:** The Demographic, Clinical, Laboratory and Radiologic Data of the Patients in this Study

	Basic Demographic Data of the three studied groups			P
	HCC (n=75)	Cirrhosis (n=75)	Control (n=75)	
Age (Years)^*^	50.12 ± 5.4	50.15 ± 5.27	50.11 ± 5.53	0.999
Male/Female^†^	60/15	59/16	61/14	0.92
Clinical, laboratory and radiologic data of the three studied groups which are significantly different at P = 0.05				
	HCC (n=75)	Cirrhosis (n=75)	Control (n=75)	P
Smoking^‡^	41 (54.7%)	12 (16%)	0 (0%)	< 0.01
Family history^‡^	24 (32%)	2 (2.7%)	--	< 0.01
Bilharziasis^‡^	36 (48%)	13 (17.3%)	--	< 0.01
Hb (g/dl)^*^	12.01±1.794 a	10.774±2.164 b	13.843±1.056 c	<0.001
Plt (x10^3^ /mm^3^)^§^	150 (130-180) a	130 (93-189) a	265 (198-320) b	<0.001
PC (%)^§^	75 (65-84) a	80 (50-995) a	95 (89-100) b	<0.001
INR^§^	1.2 (1.14-1.3) a	1.3 (1.01-1.5) a	1.04 (1-1.12) b	<0.001
BUN (mg/dl)^§^	31 (24-41) a	25 (18-63) ab	25 (19.3-30) b	0.001
Creatinine (mg/dl)^§^	0.8 (0.6-1.04) a	1 (0.7-1.47) b	0.71 (0.61-0.81) c	<0.001
Total protein (g/dl)^§^	7.2 (6.4-8) a	6.5 (6-7.1) b	7.4 (6.9-7.9) a	<0.001
Albumin (g/dl)^§^	3.1 (2.7-3.1) a	3.7 (2.6-4.2) b	4.3 (4-4.5) c	<0.001
AST (IU/l)^§^	71 (53-96) a	35 (20-73) b	19 (15-26) c	<0.001
ALT (IU/l)^§^	34 (18-52) a	29 (16-76) a	17 (10-25) b	<0.001
ALP (IU/l)^§^	102 (76-150) a	66 (33-101) b	78 (58-85) b	<0.001
TBIL (mg/dl)^§^	1.5 (0.9-2.3) a	0.9 (0.5-2.9) b	0.7 (0.5-0.9) c	<0.001
DBIL (mg/dl)^§^	0.5 (0.3-1) a	0.2 (0.1-0.9) b	0.1 (0.1-0.15) c	<0.001
AFP (ng/ml)^§^	57.7 (20-154)	7.4 (4-28)	--	<0.001
Spleen status^‡^				
Average-sized	22 (29.3%)	48 (64%)		< 0.01
Enlarged	53 (70.7%)	27 (36%)	--	
Clinical, laboratory and radiologic data of the three studied groups which are not significantly different at P = 0.05				
	HCC (n=75)	Cirrhosis (n=75)	Control (n=75)	P
Diabetes^‡^	26 (34.7%)	23 (30.7%)		0.601
Ascitis^‡^	33 (44%)	26 (34.7%)	--	0.441
Encephalopathy^‡^	12 (16%)	10 (13.4%)	--	0.833
Child Pough stage^‡^				
A	34 (45.3%)	44 (58.7%)		0.119
B	26 (34.7%)	15 (20%)		
C	15 (20%)	16 (21.3%)		
MELD score^§^	10 (8-12)	11 (7-18)		0.475
TLC (x10^3 ^cell/µl)^§^	5.6 (4.8-7.2) a	6.5 (5-8.4) a	5.9 (5-7.3) a	0.382
Liver size^‡^				
Average-sized	31 (41.3%)	43 (57.3%)	--	0.05
Enlarged	44 (58.7%)	32 (42.6%)		

^*^ Data are presented as mean ± SD; ^† ^Data are presented as number; ^‡^ Data are presented as number (Percent); ^§ ^Data are presented as median (25^th^-75^th^); Groups bearing the same initials are not statistically different at P < 0.05

**Table 2:** Frequency Distribution of MicroRNA 499 (rs3746444) Genotypes, Alleles and Odd’s Ratios among the Studied Groups

Genotypes	HCC (n=75)	Cirrhosis (n=75)	Control (n= 75)	P
AA	41 (54.7%)	41 (54.7%)	31 (41.3%)	0.009
AG	32 (42.6%)	21 (28%)	30 (40%)	
GG	2 (2.7%)	13 (17.3%)	14 (18.7%)	
AA/AG^*^	73 (97.3%)	62 (82.7%)	61 (81.3%)	0.005
GG/AG^†^	34 (45.3%)	34 (45.3%)	44 (58.7%)	0.169
Alleles	HCC (n=150)	Cirrhosis (n=150)	Control (n=150)	P
A allele	114 (76%)	103 (68.7%)	92 (61.3%)	0.024
G allele	36 (24%)	47 (31.3%)	58 (38.7%)	
Genotypes	HCC (n=75)	Cirrhosis (n=75)	OR (95% CI)	P
AA/AG^*^	73 (97.3%)	62 (82.7%)	0.131 (0.028-0.601)	0.003
Alleles	HCC (n=150)	Cirrhosis (n=150)	OR (95% CI)	P
A allele	114 (76%)	103 (68.7%)		0.165
G allele	36 (24%)	47 (31.3%)	0.692 (0.416-1.152)	
Genotypes	HCC (n=75)	Control (n=75)	OR (95% CI)	P
AA/AG^*^	73 (97.3%)	61 (81.3)	0.119 (0.026-0.847)	0.002
Alleles	HCC (n=150)	Control (n=150)	OR (95% CI)	P
A allele	114 (76%)	92 (61.3%)		0.006
G allele	36 (24%)	58 (38.7%)	0.501 (0.304-0.825)	

Data are presented as number (percent); ^*^versus GG; ^†^versus AA.

**Table 3:** Comparison between (AA+AG) and GG Genotypes of MicroRNA 499a (rs3746444) in HCC and Cirrhosis Groups as Regards the Laboratory Data

	AA+AG (n=135)	GG (n=15)	P
Hb (g/dl)	11.384 ± 2.111	11.513 ± 1.801	0.819
TLC (x10^3^ cell/µl)	6.1 (5-7.8)	5.6 (4.2-6.9)	0.158
Plt (x10^3^/mm^3^)	145 (110-180)	145 (95-230)	0.618
PC (%)	77 (60-90)	90 (50-100)	0.263
INR	1.2 (1.1-1.5)	1.1 (1-1.5)	0.307
Urea (mg/dl)	30.2 (21.4-47.1)	21.4 (18-36.4)	0.153
Creat (mg/dl)	0.86 (0.68-1.21)	0.9 (0.6-1.46)	0.985
Total protein (g/dl)	6.9 (6.2-7.8)	6.2 (6-7.2)	0.147
Albumin (g/dl)	3.2 (2.7-3.9)	3.9 (2.6-4)	0.218
AST (IU/l)	58 (35-95)	25 (18-44)	0.002
ALT (IU/l)	34 (18-60)	22 (14-29)	0.036
ALP (IU/l)	92 (62-137)	58 (19-85)	0.006
TBIL (mg/dl)	1.3 (0.7-2.4)	0.7 (0.4-1.2)	0.021
DBIL (mg/dl)	0.4 (0.2-1)	0.15 (0.1-0.3)	0.01
AFP (ng/ml)	23.8 (6-87.3)	19 (6-45)	0.573

**Table 4:** Frequency Distribution of MicroRNA 196a2 (rs11614913) Genotypes and Alleles among the Three Studied Groups

Genotypes	HCC (n=75)	Cirrhosis (n=75)	Control (n= 75)	P
CC	37 (49.3%)	42 (56%)	30 (40%)	0.344
CT	32 (42.7%)	26 (34.7%)	35 (46.7%)	
TT	6 (8%)	7 (9.3%)	10 (13.3%)	
CC/CT^*^	69 (92%)	68 (90.7%)	65 (86.7%)	0.533
TT/CT^†^	38 (50.7%)	33 (44%)	45 (60%)	0.144
Alleles	HCC (n=150)	Cirrhosis (n=150)	Control (n=150)	P
C allele	106 (70.7%)	110 (73.3%)	95 (63.3%)	0.152
T allele	44(29.3%)	40 (26.7%)	55 (36.7%)	
Genotypes	HCC (n=75)	Cirrhosis (n=75)	OR (95% CI)	P
CC/CT^*^	69 (92%)	68 (90.7%)		0.772
TT/CT^†^	38 (50.7%)	33 (44%)		0.414
Alleles	HCC (n=150)	Cirrhosis (n=150)	OR (95% CI)	P
C allele	106 (70.7%)	110 (73.3%)		0.607
T allele	44 (29.3%)	40 (26.7%)		
Genotypes	HCC (n=75)	Control (n=75)	OR (95% CI)	P
CC/CT^*^	69 (92%)	65 (86.7%)		0.290
TT/CT^†^	38 (50.7%)	45 (60%)		0.250
Alleles	HCC (n=150)	Cirrhosis (n=150)	OR (95% CI)	P
C allele	106 (70.7%)	95 (63.3%)		0.177
T allele	44 (29.3%)	55 (36.7%)		

Data are presented as number (percent); ^*^versus TT; ^†^versus CC.
